# Factors associated with students’ perceptions of role modelling

**DOI:** 10.5116/ijme.57eb.cca2

**Published:** 2016-10-14

**Authors:** Bahareh Bahman Bijari, Morteza Zare, Ali Akbar Haghdoost, Azam Bazrafshan, Amin Beigzadeh, Maryam Esmaili

**Affiliations:** 1 Department of Paediatrics, Afzalipour Hospital, Kerman University of Medical Sciences, Kerman, Iran; 2 Nutrition Research Center, School of Nutrition and Food Sciences, Shiraz University of Medical Sciences, Shiraz, Iran; 3 Regional Knowledge Hub for HIV/AIDS Surveillance, Institute for Futures Studies in Health, Kerman University of Medical Sciences, Kerman, Iran; 4 Neurosciences Research Center, Institute of Neuropharmacology, Kerman University of Medical Sciences, Kerman, Iran; 5 Research Center for Health Services Management, Institute for Futures Studies in Health, Kerman University of Medical Sciences, Kerman, Iran; 6 Research Center for Social Determinants of Health, Institute for Futures Studies in Health, Kerman University of Medical Sciences, Kerman, Iran

**Keywords:** Faculty development, humanism, medicine, pharmacy, role modelling

## Abstract

**Objectives:**

To determine which professional and humanistic attributes demonstrated by teachers in the health disciplines caused them to be perceived by students as positive or negative role models.

**Methods:**

Quantitative empirical data were gathered using a self-administered questionnaire by graduating students in medical, dentistry, and pharmacy schools at Kerman University of Medical Sciences. A total of 3 graduating cohorts, comprising about 220 students, were selected for this study. Surveys were distributed during January-March 2013.

**Results:**

In total, 183 students participated in the study. Altogether, students considered 504 and 473 academic staff as positive and negative role models (PRMs and NRMs), respectively. Women were considered more negatively than men (mean scores: -12.13 vs. -11.6, p=0.04). While clinicians were considered more positively than basic scientists (mean scores: 12.65 vs. 10.67, p=0.001), dentists received higher positive scores than physicians or pharmacists (average scores: 13.27 vs. 12.99 and 9.82). There was a significant relationship between the personality of the students and the overall characteristics of their perceived role models (β for PRMs=0.35, p<0.0001; and β for NRMs= 0.20, p= 0.039).

**Conclusions:**

Humanistic and professional attributes were proposed as major components of personal traits in perceived role models. Demonstration of humanistic attributes by teachers was strongly correlated with the students’ perception of the role models. It is suggested that the role of humanistic and professional attributes should be highlighted across medical disciplines in an effort to develop or improve role modelling by academic staff.

## Introduction

Role modelling is regarded as the primary source of learning humanistic and ethical aspects of healthcare. It is described as a cognitive process in which students actively observe and imitate the attributes of their perceived models. ^1^^,^^2^

Different theories have been proposed over the years to describe how people learn by observing the behaviour of others.^3^ within this context; Bandura’s social learning theory bridged the gap between cognitive and behavioural theories to propose a comprehensive model that could explain different learning experiences that occur in real-life situations. Bandura believed that “most human behaviour is observationally learned through modelling: from observing others, one forms an idea of how new behaviours are performed, and on later occasions this coded information serves as a guide for action”.^4^^,^^5^ According to Bandura’s theory, whether we learn from direct or vicarious experiences, most of our learning usually involves other people in a social setting. It is on the basis of our observations and interactions with other people that our conditions, including our standards for performance and for moral judgement, are developed.^4^^,^^5^

Role modelling in medical settings draws heavily on the idea that learning is generally characterized by the students' motivation to acquire medical knowledge, expertise, values, and clinical skills from the professional role models inside and outside the medical school. These professional qualities are not acquired through formal training.  Rather, in everyday practice, students focus and act like the teachers they observe to perform well in that setting.  In this context, students learn observationally from their role models not only how to speak and act as a doctor, but also how to think like a doctor.^6^

The importance of role modelling in medical education is well understood and previous studies have identified different components and typologies of role modelling.^7^^-^^11^ However, recent calls for role modelling as a widely accepted teaching method in clinical settings have highlighted ongoing concerns about how humanistic dimensions of role models affect the students’ establishment of a professional identity.^12^^,^^13^ While positive role models (PRM) are those who can be followed and emulated for their manners, and humanistic and professional characteristics, negative role models (NRM) are examples  to be avoided^14^ for their unprofessional behaviour and characteristics, poor support, uncaring behaviour toward students, and  impatience.^2^^,^^15^ Therefore, it is important to use specific criteria to distinguish between PRMs and NRMs.

It is also well understood that role modelling is an important part of the learning process,^7^^,^^8^^,^^11^^,^^15^ which can be regarded as an integral component of medical education. It is also affected by ethnic background and cultural contexts. ^16^ These factors influence the choice of role models in medical schools.^16^ Therefore, different role models and typologies could emerge in different cultural settings and even at different educational levels. In this regard, empirical studies have been undertaken to explore the attributes of role models in both medical students and residents as learners.^6^^,^^10^^,^^11^^,^^17^

Although research on the common characteristics of PRMs has grown rapidly, research specifically on NRMs remains limited and little is known of what contributes to an individual being perceived as an NRM.  In addition, little attention has been paid to study differences across disciplines outside medicine. While much research has focused on the multifaceted and complex phenomenon of role modelling in diverse medical settings, limited evidence exists on the perceptions of dental^18^^,^^19^ and pharmacy students^20^^-^^23^ about role modelling. Therefore, we examined the insights as well as demographic attributes of graduating students across health disciplines (medicine, dentistry, and pharmacy)  to determine which professional and humanistic attributes demonstrated by medical teachers, as well as other potential factors, caused them to be perceived by students as PRMs and NRMs.

## Methods

### Study design

This was a cross-sectional study conducted at Kerman University of Medical Sciences during January-March 2013. The study was approved by Kerman University of Medical Sciences Research Ethics Committee. Anonymity and confidentiality were also considered for the study.

### Participants

The study population included medical, dentistry, and pharmacy graduating students at Kerman University of Medical Sciences (a large Iranian medical university). A total of 3 graduating cohorts, comprising 220 students, were selected for the study. Surveys were distributed during January-March 2013. Of the 220 students invited to participate in the study, 183 ultimately returned the questionnaires, giving a response rate of 83%. Oral consent was obtained from all participants.

Almost half of the students were from medical school (49%) and the rest were from dentistry (29%) and pharmacy schools (22%). Over two thirds of the students were female (68%). The highest and lowest average age were found in the medical (26.3±2.3) and dentistry students (23.0±1.0).

About 58% of the students intended to continue their education beyond their current degree, and the rest were interested to work as a physician. Of the students surveyed, 24% estimated the level of their academic performance to be good while 69% estimated the level of their academic performance to be adequate.

**Figure 1:**
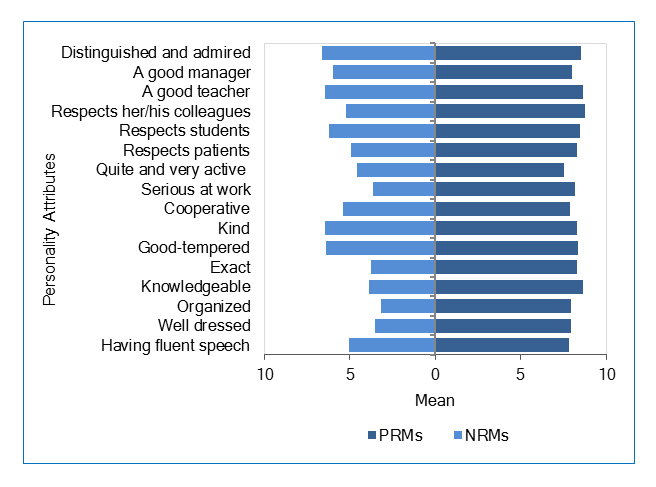
Personal attributes of PRMs and NRMs perceived by medical, dentistry, and pharmacy students (attributes ranged from -10 to 10)

**Figure 2:**
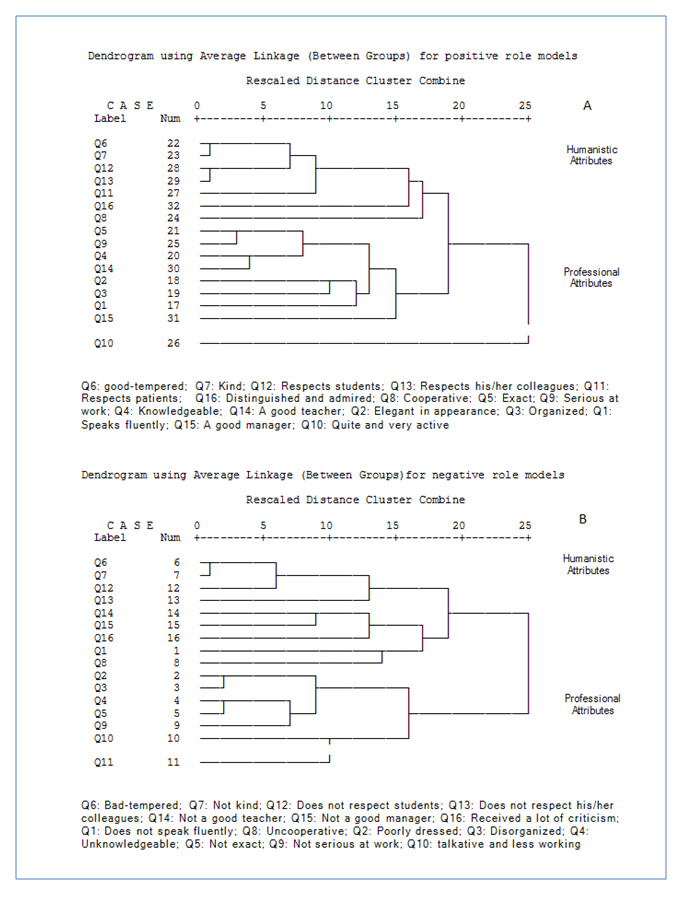
Cluster analysis of personal attributes proposed by medical, pharmacy, and dentistry students for their perceived positive (A) and negative (B) role models. Note: Shorter lines indicate stronger correlation between two variables.

### Data collection

A structured, self-administered role modelling questionnaire was developed using existing literature and findings from two quantitative studies,^11^^,^^24^ and through collaboration with three domain experts and advisors in medical education and evaluation field. According to received comments from these experts, a set of professional attributes were added to the questionnaire. Therefore, the overall content of the questionnaire covered two broad sets of humanistic and professional qualities of role models including 16 pre-specified attributes. The content of the questionnaire was then tested in a pilot study involving 15 students to assure the clarity, lack of ambiguity, and internal consistency of the scale (Cronbach’s alpha=0.83).

The questionnaire comprised generic competencies common to all disciplines and was divided into three main sections. In section 1, participants were invited to fill in their personal information, including their age, gender, and academic performance. They were also asked to rate their own characteristics according to a list of 10 pre-specified attributes (rating scale -10 to +10). In sections 2 and 3, participants were invited to name up to three PRMs and three NRMs, respectively. All participants were prompted to rate the characteristics of their perceived role models according to a list of 16 pre-specified relevant attributes.  For simplicity, participants used the letters A, B, and C on a 0 to +10 rating scale for PRMs and E, F, and G on a 0 to -10 rating scale for NRMs. A series of questions elicited relevant demographic factors for both PRMS and NRMS.

### Data analysis

The data were analysed using Stata/SE version 11.0 (Stata Corp, Texas, USA), to extract the principal attributes of perceived PRMs and NRMs. PRMs and NRMs were compared according to their demographic factors using univariate, One-way ANOVA, independent sample t-test, or Pearson correlation analysis. Hierarchical Clustering/dendrogram was used to measure the similarities among personal attributes. Multivariate linear regression was used to reduce the effect of potential confounders and to predict the overall effect of demographic attributes on the role modelling status.

## Results

### Personal characteristics of positive and negative role models

In total, 977 role models were identified among whom the frequencies of PRMs (n=504) and NRMs (n=473) was very close. One third of all reported role models were female (33%). The largest number of role models was proposed by medical students (n=399), and most role models were 40-50 years old (n=490).

For PRMs, “Respects his/her colleagues”, “Knowledgeable”, and “A good teacher” were ranked as the most important attributes. Conversely, “Not distinguished or admired”, “Not a good teacher”, and “Unkind” were ranked as the most important attributes of NRMs (Figure 1). Faculty members most likely to be seen as PRMs were those highly rated on all attributes. However, NRMs were most likely those rated poorly in personal attributes.

### Hierarchical clustering of characteristics of positive and negative role models

Cluster analysis of personal attributes in perceived PRMs and NRMs revealed similar patterns (Figure 2). It is possible to find large scale groups of personal attributes for PRMs. It was found that “Quite and very active” were completely separated from all the other attributes in PRMs. It indicates that the correlation of PRMs in this attribute is substantially different from the correlation of PRMs in the remaining attributes. The remaining attributes can be clustered into two groups. For PRMs, the first group comprised five attributes: ''Good-tempered'', "kind'', ''Respects students'', ''Respects his/her colleagues'', and ''Respects patients''. Overall, these attributes could be called humanistic qualities. The remaining attributes were clustered in the second group. This group included but was not limited to ''Exact'', ''Serious at work'', ''Knowledgeable'' and ''A good teacher''. These attributes could be called professional qualities (Figure 2).

Based on dendrogram figure (Figure 2) “Good-tempered” and “kind” were more relevant and similar to each other (r = 0.75, p<0.0001). “Respects students” and “Respects her/his colleagues” were similarly clustered (r =0.69, p<0.0001) indicating that these attributes were more similar and relevant to each other than they were to any other attributes.  Some other similar attributes could be found in lower similarity degrees by looking at Figure 2. “Exact” and “Serious at work” were among such relevant attributes (r = 0.66, p<0.0001).

Similar analysis for NRMs revealed two large scale groups of personal attributes. Two humanistic attributes, ''Bad- tempered'' and ‘‘Unkind’’, were the most similar and relevant attributes to each other than to any other attributes (r = 0.76, p<0.0001). Several professional attributes, “Poorly dressed” and ''Disorganized'' (r =0.71, p<0.0001), as well as “Unknowledgeable” and “Not exact” (r = 0.68, p<0.0001), were the other similar attributes clustered in two other groups.

### Multivariate analysis of PRM and NRM

Further inspection of the role modelling data revealed that age as a fixed linear effect was not statistically significant across either PRMs (p=0.840) or NRMs (p=0.150). This means that age of academic staff did not have any association with their positive or negative reputations.  In addition, role models did not seem to differ significantly with respect to their gender (PRMs= -0.77, p=0.182; and NRMs=-1.93, p=0.045,). Clinicians received higher adjusted scores than basic scientists (PRMs= 2.51, p=0.001; NRMs=2.25, p=0.057). In addition, the overall adjusted characteristics of perceived role models according to disciplines differed significantly. In both PRM and NRM groups, models perceived by dentistry students received higher adjusted scores (13.27, -13.63 for PRMs and NRMs, respectively) compared to medical and pharmacy. There was a significant relationship between the personality of students and the overall characteristics of their perceived role models (β for PRMs=0.35, p<0.0001; and β for NRMs= 0.20, p= 0.039) (Table 1).

**Table 1:** Prediction of PRM and NRM scores according to multivariate linear regression analysis

Variables			PRM				NRM			
			β*		p value		β*		p value	
Specialty										
	Dentistry		Ref.		--		Ref.		--	
	Medicine		-0.64		0.328		-2.13		0.048	
	Pharmacy		-3.61		<0.0001		-2.99		0.045	
Gender (Student)										
	Female		Ref.		--		Ref.		--	
	Male		-0.77		0.182		-1.93		0.045	
Gender (Role Model)										
	Female		Ref.		--		Ref.		--	
	Male		-0.40		0.495		1.43		.142	
Field										
	Basic Science		Ref.		--		Ref.		--	
	Clinical Science		2.51		0.001		2.25		0.057	
Degree										
	Instructor		Ref.		--		Ref.		--	
	Assistant Professor		0.81		2.627		-0.31		0.856	
	Associate Professor		0.42		0.801		2.66		0.136	
	Full Professor		1.59		0.364		2.89		0.176	
Students Personality			0.35		<0.00001		0.20		0.039	

*β is the regression coefficient that shows the difference between the value in each subgroup versus the value in the reference subgroup. Positive values for a subgroup of PRM or NRM (such as field) indicated higher values for PRMs and NRMs in clinical sciences rather than basic sciences. This implied that clinical role models received higher scores than basic science role models.

## Discussion

This study presents empirical evidence that supports the importance of both humanistic and professional qualities in the identification of PRMs and NRMs by a wide range of medical, dental, and pharmacy students. Faculty members most likely to be seen as PRMs were those highly rated in all humanistic and professional qualities. However, NRMs were most likely those rated poorly in either humanistic or professional qualities. The overall characteristics of PRMs and NRMs were significantly different among disciplines but mostly identical in terms of demographic factors.

In this survey of students’ opinions regarding their perceived PRMs and NRMs, all students reported to have observed NRMs as well as PRMs almost frequently. Many faculty members did not exhibit behaviours and skills that graduating students perceived as characteristics of PRMs. In this study, faculty members who emphasized the humanistic behaviours regarding students and patients were more likely to be perceived as PRMs. Earlier works with students and residents indicated that those who were perceived to be uncaring towards patients, or to demonstrate unprofessional attitudes or unprofessional behaviour or disrespect for students were judged as NRMs.^15^  The current study seems to confirm earlier findings that more than half of faculty members were perceived as NRMs.^25^  There is a great body of literature regarding the lack of good role models in diverse clinical settings.^26^

Previous research has shown that many clinical teachers were frequently observed as poor role models by medical students and junior doctors.^27^ In one study, one third of the residents and half of the clinical clerks reported that their clinical teachers were not PRMs for doctor-patient relationships.^28^ This may in part be due to the fact that teachers may have a limited understanding of their strengths and weaknesses as clinical teachers, of students’ perception of their humanistic dimensions and behaviours, and of the potential for developing their teaching skills, and possibly their effectiveness as role models.^29^ Since role models have an important effect on students’ career choice, character formation, and professional identity, our findings should concern us and the academic institutions about the unfavourable outcomes of the lack of good role models in the development of professionalism, improvement in health, and the quality of medical education.

The good news is that strong empirical support exists for the perception of both PRMs and NRMs as learning strategies,^2^^,^^15^^,^^16^^,^^30^ and “character formation” of medical students.^31^ While PRMs offer the individual role-expectation,^2^ what is role-expectation? skill expertise, and performance standards, NRMs  represent behaviours and attitudes that the students seek to avoid,^2^  and by doing so, students learn how not to behave in a particular context.^5^ As NRMs can provide learning opportunities for students, it is important that students learn to distinguish between NRMs and PRMs.^15^

Besides, teachers should be informed about their role modelling status and their current impression on the students. This could help teachers explore their reflections more actively and to improve or modify their behaviour according to the students’ evaluations. It is suggested that improving role modelling at an individual level would require the faculty to be aware of their role modelling status and performance, to reflect upon their experiences, and to participate in staff development when necessary.

The present findings suggest that faculty most likely to be seen as PRMs were those highly rated on all humanistic and professional qualities. However, NRMs were most likely those rated poorly in either humanistic or professional qualities. This could be partly associated with the halo effect. Students, who perceived a teacher as a PRM, had a tendency to rate all attributes or statements in the evaluation questionnaire high and with little variation. When students perceived a teacher as an NRM, they seemed to be searching through the questions or attributes more carefully, looking for a way to find why the teacher was unfavourable.^32^ Studies examining halo effects in other situations have shown mixed results^33^ and few studies have looked at the role of halo effects on the students’ perception of role modelling and their evaluations of humanistic and professional attributes related to PRMs and NRMs. Consequently, key questions regarding the relative importance of halo effects in role modelling remain unanswered and require further investigations.

Our findings indicate meaningful correlations within personal attributes; it is, therefore, possible to improve personal by strengthening and improving some attributes.  As many of the humanistic and positive attributes represent behaviours that could be modified or skills that could be acquired, the present findings also suggest that one solution for this problem will be to initiate some efforts to develop or improve role modelling by highlighting the role of humanistic attributes in clinical teachers across disciplines.

It seems that humanistic attributes have more impression on the students’ perceptions for role modelling. PRMs are perceived as those rated favourably and distinctly higher in almost all humanistic and professional attributes. This may imply that becoming a PRM requires a substantial development in all aspects of humanistic and professional attributes. However, having any unfavourable attribute or unprofessional manner might lead to being perceived as an NRM by students.

Primary among the unexpected findings in this study was the failure to provide evidence on the effect of gender on the students’ perception of role models, which was contrary to the results reported by previous studies.^2^^,^^34^^-^^36^ our findings suggest that female role models are less frequently reported and female students significantly reported male models particularly in PRMs. This may in part be due to the fact that female faculty comprises little share of medical faculty and therefore females are less frequently observed and followed by students. Our findings also confirm that women typically report fewer role models who match them in terms of gender and thus they are expected to translate male role models’ behaviour into one that works for them.2 In this sense, the lack of female professionals and role models has been also proposed as a substantial barrier to the females’ achievements and career development.^37^^,^^38^

Our results also show that the characteristics of perceived PRMs and NRMs were weakly associated with the characteristics of students. This finding demonstrates that a student’s personality is less likely relevant to his/her perceptions of role modelling. While a few studies have focused on the effect of students’ personality on their evaluations of teaching (SETs),^39^^,^^40^ the role of students’ personality on their perception of role modelling remains unclear. Further investigations are needed to study role modelling in terms of personality groups in students and their perceived role models.

Similar to the findings reported in previous studies,^25^^,^^34^we found significant association between the characteristics of role models and disciplines in PRM and NRM groups. This may be partly due to the different context of medical, pharmacy, and dentistry curriculums at the undergraduate level. However, our understanding of role modelling in pharmacy and dentistry students is limited (due to lack of conclusive evidence) and further investigations are needed to better understand pros and cons of role modelling in undergraduate fields such as pharmacy and dentistry.

Some limitations of this study should be considered when interpreting our results. First, we studied the insights of graduating students about teachers perceived as role models.  Other types of role modelling, such as that by physician or resident role models, may exist and were not considered in this study. Second, this study relied on self-reporting.  Therefore, the actual context from which students’ opinions were drawn is unknown. Future direct observation of role models could address this limitation of the current study.

## Conclusions

Our findings suggest that the teachers’ demonstration of humanistic attributes and the students’ perception of role modelling were strongly related. Many of these positive attributes represent behaviours that could be modified or skills that could be acquired. It is, therefore, suggested that academic institutions make efforts to develop or improve the role modelling of faculty by highlighting the role of humanistic attributes in clinical teachers across disciplines.  As medical education is about “learning to be a doctor by being a doctor; caring for patients under controlled conditions of safety and care”,^41^ teachers are required to be aware of learners’ opinions and be prepared to recognize and respond to the variety of needs about professional and humanistic outcomes of their teaching. Institutions are required to support faculty development activities to present students with a wide range of role models such as researcher, specialist, or teacher. according to their disciplines and diverse expectations and needs.

### Acknowledgements

The authors wish to acknowledge the work of Fardad Firooznia and Roghaie Ilghami in improving the quality and language of this paper and to thank them for their support. Funding/ Support: This work was a part of fellowship thesis in Medical Education in Kerman University of Medical Sciences, Kerman, Iran.

### Conflict of Interest

The authors declare that they have no conflict of interest.
